# Preparation and Characterization of Flexible Substrate Material from Phenyl-Thiophene-2-Carbaldehyde Compound

**DOI:** 10.3390/ma9050358

**Published:** 2016-05-11

**Authors:** Ashiqur Rahman, Mohammad Tariqul Islam, Md Samsuzzaman, Mandeep Jit Singh, Md. Akhtaruzzaman

**Affiliations:** 1 Department of Electrical, Electronic and Systems Engineering, Faculty of Engineering and Built Environment, Universiti Kebangsaan Malaysia, Bangi 43600, Malaysia; ashiqur@siswa.ukm.edu.my (A.R.); mandeep@eng.ukm.edu.my (M.J.S.); 2 Space Science Centre (ANGKASA), University Kebangsaan Malaysia, Bangi 43600, Malaysia; 3 Department of Computer and Communication Engineering, Faculty of Computer Science and Engineering, Patuakhali Science and Technology University, Patuakhali 8602, Bangladesh; sobuz@pstu.ac.bd; 4 Solar Energy Research Institute, University Kebangsaan Malaysia, Bangi 43600, Malaysia

**Keywords:** flexible substrate, microwave dielectric antenna, optical properties, organic material

## Abstract

In this paper, a novel phenyl-thiophene-2-carbaldehyde compound-based flexible substrate material has been presented. Optical and microwave characterization of the proposed material are done to confirm the applicability of the proposed material as a substrate. The results obtained in this work show that the phenyl-thiophene-2-carbaldehyde consists of a dielectric constant of 3.03, loss tangent of 0.003, and an optical bandgap of 3.24 eV. The proposed material is analyzed using commercially available EM simulation software and validated by the experimental analysis of the flexible substrate. The fabricated substrate also shows significant mechanical flexibility and light weight. The radiating copper patch deposited on the proposed material substrate incorporated with partial ground plane and microstrip feeding technique shows an effective impedance bandwidth of 3.8 GHz. It also confirms an averaged radiation efficiency of 81% throughout the frequency band of 5.4–9.2 GHz.

## 1. Introduction

Improvements in several areas of materials science have resulted in a variety of new materials with strong potential applications to microwave and millimeter-wave components. The flexible and low dielectric permittivity with low dielectric losses are the desired properties of material which can be applied in the flexible micro- and mm-wave devices. In recent years flexible materials have earned immense interest in microwave antenna technology [1,2,3], however, there are very limited analyses on the impact of bending structures. Over the last few years, the requirements of data rate increased in an unparalleled way, driven by the tremendous growth of a hyper-connected society, industrial development, and the cumulative level of interest of humans. Data traffic is supposed to be increased a thousand-fold compared to the current traffic by 2020. The emerging microwave frequency band, above 6 GHz, is one of the promising prospects to meet the challenge of a bandwidth shortage in the S band (2–4 GHz) and to motivate significant research on future technologies for wireless communication. There are also numerous wireless services that operate over a wide frequency range and demand different operating electrical characteristics, e.g., Global System for Mobile (GSM), Global Positioning System (GPS), Universal Mobile Telecommunications System (UMTS), Wireless Local Area Network (WLAN), Bluetooth, WiMAX, and others [4].

**Figure 1:**
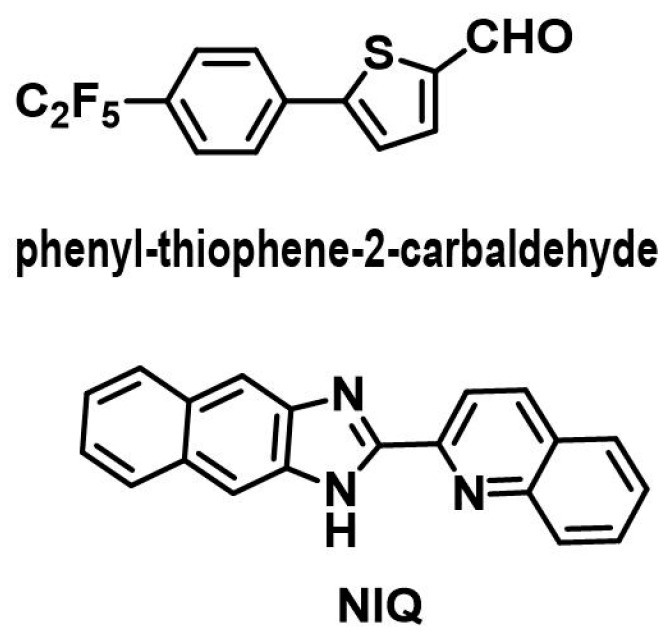
Chemical structure of compound Ak10 and NIQ.

Several researches had been done to enhance the performances, such as bandwidth, efficiency, and gain of the patch antenna by modifying the geometry of the radiating patch, such as Coplanar Waveguide(CPW)-fed [5], S-shaped slot [6], folded patch [7], hybrid dielectric resonator [8], and inverted-F antenna [9]. Moreover, material-based antennas, such as magneto-dielectric composite [10], graded composite [11], dielectric resonator antennas, and microwave dielectric ceramics [12,13,14,15] also grabbed immense interest to improve the patch antenna’s performances in recent years. However, microstrip patch antennas fabricated from aforementioned materials improved the performances with the cost of high volume, structural complexity, and cost of fabrication, high loss tangent, and mechanical rigidness. Moreover, a conventional patch antenna, consisting of full ground plane, exhibits a directional radiation pattern with narrow bandwidth. By introducing a partial ground plane in the microstrip patch antenna researchers achieved wider bandwidth. However, this microstrip patch antenna with a partial ground plane exhibits an omnidirectional radiation pattern which is similar to the monopole antenna’s radiation pattern behavior [16,17,18]. In contrast, organic materials offer low dielectric materials with a very low loss tangent, mechanical flexibility, natural degradability, simple in structure, and ease of fabrication. Therefore, organic semiconductors (small molecule/polymers) draw huge attention in the current commercial electronics markets, as well as fundamental research in the area of organic light-emitting diodes (OLEDs), organic field-effect transistors (OFETs), organic photovoltaic devices, sensors, and biological applications [19]. However, there is very little research that has been conducted on organic semiconductors for microstrip patch antenna applications. Most of current research in this area have been focused on ceramic-type materials, liquid crystal polymers (LCP), and conductive polymers [20]. To date, there is no work published yet on the fabrication process of microstrip patch antennas based on small-molecule organic substrate, except a 2-(naphtha[3.4]imidazole-2-yl) quinolone (NIQ) compound which was reported by Cherng *et al*. in 2007 (Figure 1) [21]. It showed a high loss tangent and low dielectric coefficient in NIQ thin film.

In this experiment, a new material substrate is proposed as a dielectric substrate for microwave circuitry. For proof of concept, an antenna prototype has been designed and fabricated on a novel substrate compound (Figure 1) by a magnetron sputtering technique, operating at 7 GHz. The proposed antenna, with an operating frequency range from 5.4 to 9.2 GHz, achieved more than 81% measured radiation efficiency along with a total realized gain 3.82 dBi at the center frequency, 7 GHz. Moreover, its mechanical flexibility and dielectric properties indicate this proposed antenna as a promising candidate for any flexible microwave application. Overall, the measured results are in good agreement with the simulations.

## 2. Synthesis of the Proposed Material Substrate

The compound 4-(Pentafluroethynyl)phenyl-thiophene-2-carbaldehyde was synthesized followed by Pd(PPh_3_)_4_-catalyzed Suzukki coupling reaction from 1-bromo-4-(pentafluoroethyl)benzene (1) with corresponding 5-formyl-2-thiophene broronic acid (2) in high yields as light yellow solid. The compound was purified by silica gel column chromatography (60N spherical, neutral, 40–100 mm) using a mixture solvent of dichoromethane and hexane (1:1) as eluent and the molecule structure was confirmed by ^1^H NMR (BRUKER 400 MHz), ^13^C NMR (BRUKER 400 MHz), and mass spectrometry. All other reagents and solvents are commercially available and were used without further purification unless otherwise noted. ^1^H NMR spectra are reported as follows: chemical shift in ppm (*δ*) relative to the chemical shift of Acettone-D_6_ (CD_3_COCD_3_) at 2.05 ppm, integration, multiplicities (s = Singlet, d = Doublet, m = Multiple): *δ* 10.01 (s, 1H), 8.09–8.04 (m, 3H) and 7.85–7.82 (m, 3H); ^13^C NMR (400 MHz, CD_3_COCD_3_) spectra are reported in ppm (*δ*) relative to the chemical shift of acetone-d_6_ (CD_3_COCD_3_) at 29.9 ppm and the chemical shifts of AK01 are: *δ* 126.5, 126.9, 127.4, 137.0, 138.0, 144.1, 150.5, 183.2, 205.3. The *m*/*z*: Calcd for C_13_H_7_F_5_OS 306.01; found: 306.0.

A substrate plays an important role in designing microwave devices, such as antenna prototypes. The following key parameters such as dielectric constant, loss tangent and the substrate thickness need to be considered during the selection of a substrate. The dielectric constant value has an impact on the dimension of the antenna, whereas a low loss tangent controls the antenna efficiency by reducing the radiation losses. By employing a thin and low dielectric material, radiation loss can be reduced by the open structure where the EM waves are mostly restrained within the dielectric. The proposed antenna consists of a rectangular patch printed on one side of the proposed material substrate with a partial ground plane on the other side. A quasi TEM mode of propagation was created by the absence of an upper ground plane and the dielectric above the strip. Prior to the antenna application, we investigated the optical and dielectric properties of the materials which are described in the following section.

## 3. Optical Analysis

The determination of bandgap in materials is important to obtain the basic solid state physics. The band gap is related to the electric conductivity of the materials. The optical properties of the prepared sample nanoparticles are investigated by UV-VIS reflectance spectroscopy. Optical studies include the measurements of the absorption, excitonic photoluminescence, and reflection spectra. The energy of the exciton reflection spectrum, which presents a dispersion curve, is associated with the formation of free exciton and determines the optical band gap of the semiconductor. The original spectra, in the form of plots of diffuse reflectance (*R*) against photon energy, are given by Wood (I97I) [22]. They were converted to the plots of the Kubelka-Munk function *F*(*R*) against wavelength (*λ* in nm) and photon energy (*hν* in eV). The reflection probe measures the diffuse reflectance (light scattered to all angles) as a function of wavelength in a close approximation of the signal obtained from an Integrating Sphere system [23]. The Kubelka-Munk function, *F*(*R*), allows the optical absorbance of a sample to be approximated from its reflectance according to the following equation [24]:

*F*(*R*) = (*1 − R*)^2^/*2R* (1)

**Figure 2:**
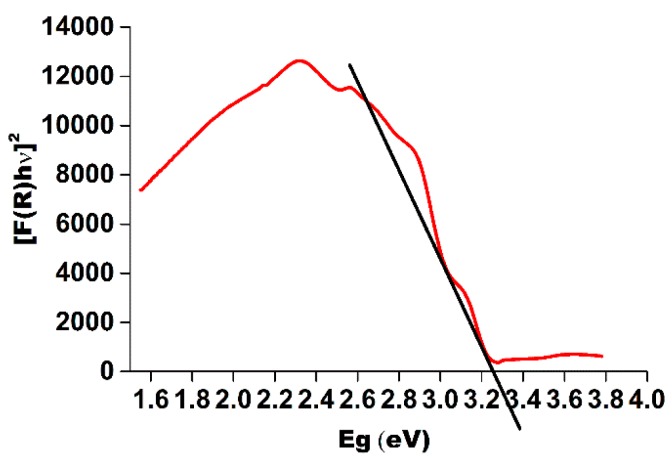
Tauc plot to determine optical bandgap from UV-VIS reflectance spectra.

For a semiconductor sample, this function allows the construction of a Tauc Plot; [*F*(*R*).*hν*]*^n^* *vs*. *hν*. For a direct band gap semiconductor the plot *n* = 2, will show a linear Tauc region just above the optical absorption edge. Extrapolation of this line to the photon energy axis yields the semiconductor band gap of 3.24 eV as shown in Figure 2.

Among all the parameters of the material, the dielectric constant (*ε_r_*) has a significant impact on antenna design. Accurate characterization of the material dielectric properties at microwave frequencies provides important information that is needed for material and circuit design, research, modeling, and quality control. A material can be categorized as dielectric, if the material store energy with the applied external electric field. Microwave dielectric materials with high *ε_r_* greatly reduces the antenna size but, on the other hand, high dielectric constant causes cross-coupling with the conductors and increases the time for electronic signal transition which exhibits high dielectric losses [25]. In addition, a low dielectric loss is usually associated with low *ε_r_* of the dielectric materials, reported by many researchers [26]. Therefore, the suitable value of the dielectric constant of the substrate is a prerequisite to bring out the best performance of the material substrate.

## 4. Microwave Characterization

**Figure 3:**
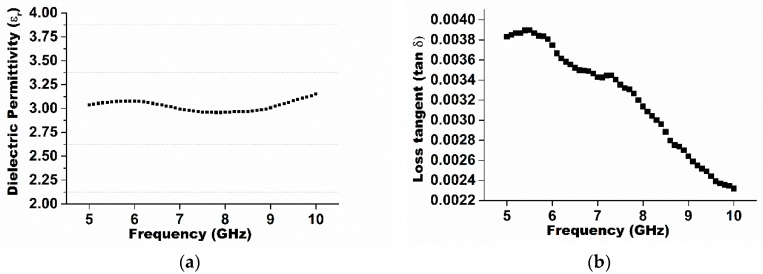
(**a**) Dielectric permittivity and (**b**) loss tangent plotted graph for the sample with the applied frequency.

The dielectric constant and loss tangent were measured through a dielectric probe kit (DAK 3.5) connected with the network analyzer (Agilent Technologies 85070E) in the frequency range from 5 to 9 GHz at room temperature. Measurements are made by simply touching the probe on the solid ceramic substrate. Measurements were non-destructive and can be made in real-time. The complete system is based on a network analyzer, which measures the material’s response to RF or microwave energy. The probe transmits a signal into the material under test (MUT). In Figure 3, the frequency is plotted as a function of dielectric permittivity, *ε_r_*. From this plot, the value of *ε_r_* is 3.03. A steady decrease in the loss tangent was observed with an applied frequency in the range from 5 to 9 GHz in Figure 3b, which can be understood on the basis of the Maxwell–Wagner model of interfacial polarization and Koop’s phenomenological theory [27,28]. According to the Debye equation, the decrease in loss tangent with the increasing applied frequency can be explained by the presence of interfacial polarization, which gives rise to a relaxation process with a long relaxation time compared to the electronic or dipolar polarization [29].

The quality factor, bandwidth, and efficiency are known as figures-of-merit of an antenna, which are interrelated and not possible to optimize individually. The quality factor is the representation of the antenna losses that consist of radiation (*Q_rad_*), conduction (*Q_c_*), dielectric (*Q_d_*), and surface wave (*Q_sw_*) losses. For a very thin substrate (*h << λ*_0_), the losses due to surface waves are very small and can be neglected. The quality factor for other losses can be expressed as [30]:

(2) $$Q_{c}=h\sqrt{\pi f\mu \sigma }$$

(3) $$Q_{d}=\frac{1}{\tan\delta }$$

(4) $$Q_{rad}=\frac{2\omega \varepsilon_{r}}{hG_{t}/l\;}K$$

where *tanδ* is the loss tangent of the substrate material, *σ* is the conductivity of the conductor associated with the patch, and ground plane *G_t_*/*l* is the total conductance per unit length of the radiating aperture. The quality factor was determined from the material dielectric loss using the following equation at resonant frequency [31]:

(5) $$Q\;\times \;f=\frac{f_{r}}{\tan\delta }$$

where *f_r_* is the resonant frequency and tan*δ* is the loss tangent. This technique is based on the measurement of the dielectric loss as a function of frequency. Moreover, lattice vibrational modes, porosity secondary phases, lattice defects, crystallizability, and inner stress of the material also influence the value of *Q × f* [32]. A low loss tangent reduces the dielectric loss and, therefore, increases the efficiency of the antenna.

## 5. Design and Performance Analysis of the Flexible Substrate Material

In order to validate the design and simulation results, the antenna was fabricated and experimentally characterized. The patch antenna constructed on proposed material substrate had been designed and analyzed using a full-wave three-dimensional high-frequency electromagnetic simulator based on the widely used finite element method. To achieve accurate patterning of the structure, at first the powder was mixed with the polyvinyl alcohol (PVA) solution, which worked as a binder. After that, the solution was dried in a hot plate at 70 °C for six hours until the flexible substrate had been achieved. Next, the copper was implemented on the substrate as a radiating element by the magnetron sputtering technique. An appropriate height and loss tangent of the substrate helped to obtain high gain and efficiency. The thin dielectric layer of 1.2 mm helped to reduce the weight as well as able to decrease the surface wave losses.

Thin substrates with lower dielectric constants are desirable for good antenna performance because they minimize undesired radiation and coupling and provide better efficiency and larger bandwidth, respectively. The width, (*w_f_*) of the microstrip transmission line and the height (*h*) of the microwave substrate have a major correlation with the characteristics impedance along with the effective relative permittivity of the air substrate microstrip system according to the following equation [33]:

(6) $$\varepsilon_{eff\;}\left(f\right)=\varepsilon_{r}-\frac{\varepsilon_{r}-\varepsilon_{es}}{1+G{\left(\frac{f}{f_{d}}\right)}^{2}}$$

where the constant:

(7) $$f_{d}=\frac{Z_{c}}{2\mu_{0}h}G=0.6+0.0009Z_{c}$$

The characteristics impedance [33]:

(8) $$Z_{c}≅\frac{1}{2\pi }\sqrt{\frac{\mu_{0}}{\varepsilon_{es}\varepsilon_{0}}}\log F_{1\;}\frac{h}{w}+\sqrt{1+{\left(2\frac{h}{w}\right)}^{2}}$$

where *F_1_* = 6 + (2*π* − 6) exp [−30.666 *h*/*w*]0.7528 and the electrostatic relative permittivity is [33]:

(9) $$\varepsilon_{es}≅\frac{\varepsilon_{r\;}+1}{2}+\left(\frac{\varepsilon_{r}-1}{2}\right){\left[1+10\left(\frac{h}{w}\right)\right]}^{-\text{ab}}$$

where, $a=1+\frac{1}{49}\log\left[\frac{{\left(\frac{w}{h}\right)}^{2}+{\left(\frac{w}{52h}\right)}^{2}}{{\left(\frac{w}{h}\right)}^{4}+0.432}\right]+\frac{1}{18.7}\log\;[1+{\left(\frac{1}{18.1}\frac{w}{h}\right)}^{3}]$ and $b=0.564{\left(\frac{\varepsilon_{r}-0.9}{\varepsilon_{r}+3.0}\right)}^{0.053}$.

The dimensions of the patch play important roles in the antenna design. The width, *W*, of the patch depends on the resonant frequency and dielectric permittivity according to the following equation [29]:

(10) $$W=\frac{c}{2f_{r}\sqrt{\frac{\varepsilon_{r}+1}{2}}}$$

where *c* is the speed of light in a vacuum and *f_r_* is the operating frequency. The patch length, *L*, regulates the resonant frequency and is a crucial design parameter. The effective length, *L_eff_*, can be calculated from the following formula [29]:

(11) $$L_{eff}=\frac{c}{2f_{r}\sqrt{\varepsilon_{reff}}}$$

The effective relative dielectric permittivity is given by [29]:

(12) $$\varepsilon_{reff}=\frac{\varepsilon_{r}+1}{2}+\frac{\varepsilon_{r}-1}{2}{\left[1+12\frac{h}{W}\right]}^{\frac{1}{2}}$$

where *h* is the thickness of the substrate. An additional line length (Δ*L*) needs to be considered on the either side of the patch because of the effect of the fringing field; this can be calculated as [29]:

(13) $$\Delta L=0.412h\frac{\left(\varepsilon_{reff}+0.3\right)\left(\frac{W}{h}+0.264\right)}{\left(\varepsilon_{reff}-0.258\right)\left(\frac{W}{h}+0.8\right)}$$

The patch length can be obtained from the following equation [29]:

(14) $$L=L_{eff}-2\Delta L$$

Finally, the resonant frequency has been calculated using the new effective length and effective relative dielectric permittivity, as shown in equation [29]:

(15) $$f_{r}=\frac{c_{0}}{2\left(L_{eff}\right)\sqrt{\varepsilon_{reff}}}$$

Equation (15) indicates that the resonant frequency is inversely proportional to the permittivity, which means decreasing the permittivity increases the resonant frequency of the antenna. A simple, miniaturized, low-loss antenna can be prototyped with this substrate, without any complexity to the metal patch by introducing a slot for wideband, or by designing a large array of patches for the high gain. Hence, a microwave dielectric material, with a thickness of 1.2 mm, permittivity (*ε_r_*) of 3.03 and a loss tangent (tan*δ*) of 0.003 had been chosen as an antenna substrate.

**Figure 4:**
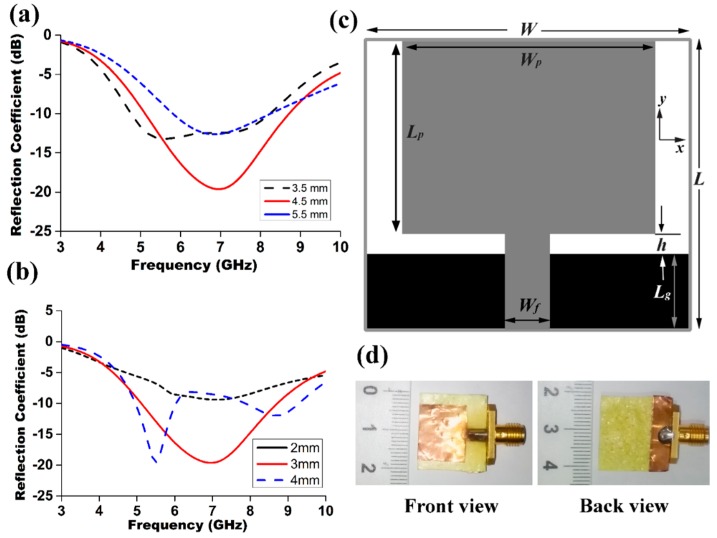
(**a**) Simulated S parameters for different ground plane size (*W* × *Lg*); (**b**) simulated S parameters for different feeding width, *W_f_*; (**c**) geometry of the proposed antenna; and (**d**) photograph of the realized antenna. Front view and back View.

**Figure 5:**
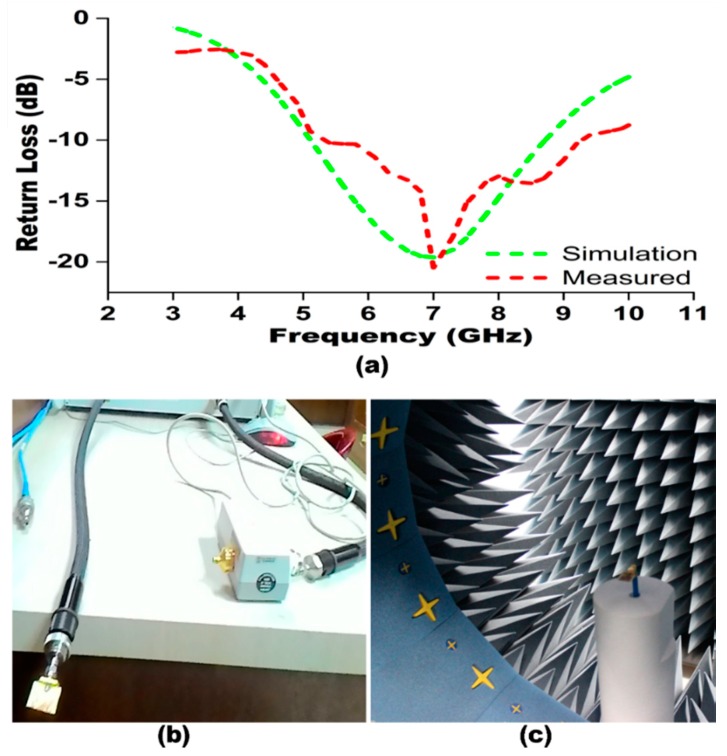
(**a**) Simulated and measured reflection coefficient of the fabricated antenna; (**b**) antenna measurement setup in PNA network analyzer; and (**c**) near-field measurement setup in Satimo.

To enhance the impedance bandwidth two parameters, a width (*w_f_*) of the microstrip line and the gap between the transmission line and the ground plane (*h*) play a significant role. It is found that a good impedance matching can be obtained by enhancing the coupling between the ground plane and the feed line [17]. Figure 4a illustrates that, an optimized value of 20 × 4.5 mm^2^ of the ground plane gives a widest impedance bandwidth with better return loss values and maintains a gap of 1.5 mm. The bandwidth also decreases beyond the certain value whether the width of microstrip feed line increases or decreases as shown in Figure 4b. A feeding width of 3 mm is the best fitting to give the widest impedance bandwidth. Figure 4c,d illustrates the geometry and the photograph of the proposed antenna with an optimum dimension of 20 × 18 × 1.2 mm^3^. The input impedance characteristics were measured by using Agilent N5227A PNA network analyzer, while a Satimo near-field anechoic chamber was used to evaluate the antenna performances, such as gain, efficiency, and radiation pattern as shown in Figure 5b,c. This system calculates the electric fields within the near field of the antenna which is equivalent to the far field data of the antenna under test by using near-field measurement techniques. The electric charge and the electromagnetic induction effects strongly occur in this near field region, which also known as the Fresnel region. This region is commonly given by [30]:

(16) $$0.62\sqrt{\frac{D^{3}}{\lambda }}<R<\frac{2D^{2}}{\lambda }$$

**Figure 6:**
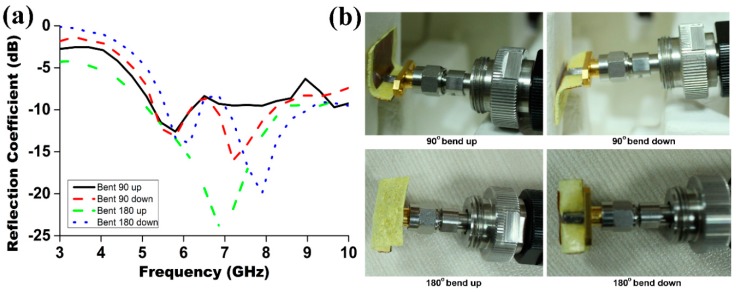
(**a**) Measured reflection coefficient of the proposed antenna under various bending condition; and (**b**) the antenna in various bending positions: 90° bending upward; 90° bending downward; 180° bending upward; 180° bending downward.

These effects fade out more rapidly than the far field with increasing distance from the antenna. Later, a Fourier transformation is used to calculate the equivalent far field data for this near-field measured data. The antenna, mounted on the test board, was positioned in the center of a circular arch that consist of 16 separate measurement probes. These probes were spaced equally apart along the circular surface. The antenna was rotated horizontally through 360°, and the combination of this rotation and the array of the probes allowed a full 3D scan of the antenna to be carried out, allowing the full 3D radiation pattern to be measured, plotted, and analyzed. Information about antenna gain and efficiency can be calculated afterwards from the far-field radiation pattern data. Figure 5a illustrates the plots of the reflection coefficient (S_11_) for both simulation and measurement of the proposed antenna. It is observed from the plot that the prototyped antenna achieved a good impedance matching from 5.4 to 9.2 GHz with a measured |S_11_| parameter below −10 dB. The return loss was reduced to a highest value of 19.62 dB and 20.47 dB for the simulation and measured results, respectively. The simulation and measured operating bandwidth of the proposed antenna were found to be 3.5 GHz and 3.8 GHz with the resonant frequency at 7.0 GHz. The material exhibits remarkable flexibility and robustness, consequently without any mechanical damage the antenna can be bent upward and downward both at ±90° and ±180° as shown in Figure 6b. The reflection coefficient against the frequency for the antenna bending at ±90° and ±180° were illustrated in Figure 6a. Such bending experiments were carried out from the practical interest to demonstrate the flexibility of this antenna. From the figure it was observed that for all the cases the antenna resonance frequency was within the operating band, but with lower impedance bandwidth.

**Figure 7:**
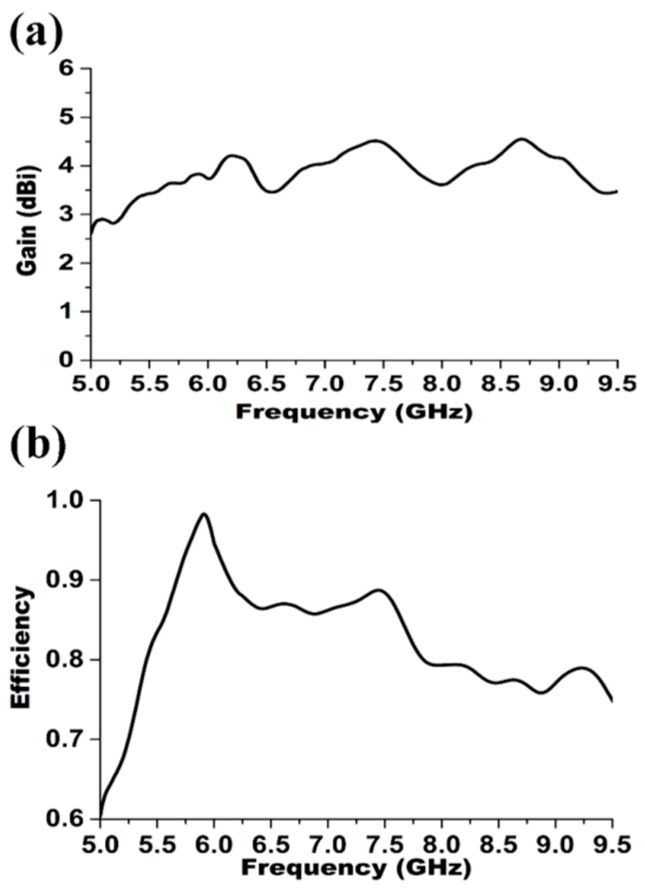
(**a**) Measured peak antenna gain of the realized antenna and (**b**) measured antenna efficiency of the proposed antenna.

The realized peak gain of the prototyped antenna is depicted in the Figure 7a. It is seen that the proposed antenna has an average peak gain of 3.78 dBi. The maximum realized gain is 4.99 dBi at 6.2 GHz and the measured gain variations are less than ±1.21 dBi. The total antenna efficiency (*ηT*), besides the internal losses of the antenna structure, also includes matching losses at the input port defined as [18]:

(17) $$\eta T=\eta R\left[1-│\Gamma {│}^{2}\right]$$

where *Γ* is the reflection coefficient at the input port and *ηR* is the radiation efficiency that can be defined in terms of radiated power (*P_R_*) and power loss in the antenna (*P_L_*) structure [18]:

(18) $$\eta R=\frac{P_{R}}{\left(P_{R}+P_{L}\right)}$$

**Figure 8:**
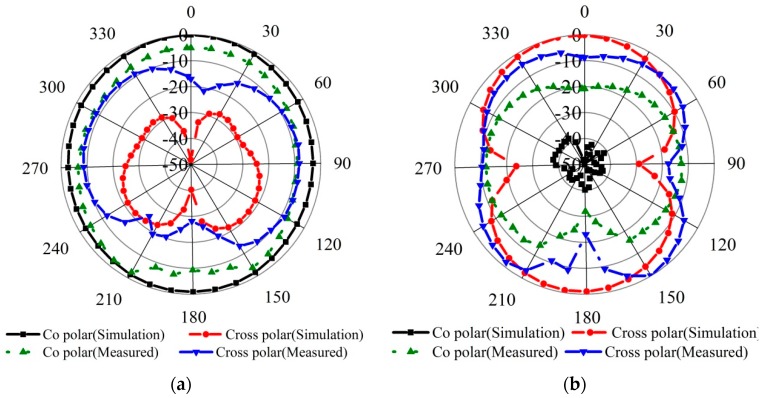
Radiation patterns in 2D view of the (**a**) *E*-plane and (**b**) *H*-plane at 7 GHz.

The power loss can be further divided into losses due to the metallic antenna parts and dielectric. Thus, the maximum total antenna efficiencies are nearly equal to the maximum radiation efficiencies. The efficiency measurement was carried out using a Satimo near-field measurement system, and is presented in Figure 7b. The result shows that the peak total efficiency is 99.2% with an average of 81% across the operating bandwidth from 5.4 to 9.2 GHz. Figure 8a–d display the simulated and measured radiation patterns of the proposed antenna at 7.0 GHz in two principal planes-namely *H*- plane and *E*-plane in 2D view, normalized to the maximum gain. It is observed that co-polarized field of the radiation patterns are omnidirectional in *H*-plane, whereas the cross-polarization resembles a donut shape created by two nulls at *θ* = 0° and *θ* = 180°. A 360° coverage signal in all directions (horizontally) can be provided by this antenna for two-way communication. In the *E*-plane two nulls are observed in the broadside direction that is similar to the typical monopole antennas for copolarization field. An omnidirectional signal level in the *H*-plane for cross-polarization has been formed that is directed at the vertical plane, which determines the point of the strongest signal by avoiding the signal redundancy and interference from the other antennas radiating at the same plane. Some dips can be observed both in the *E*-plane and *H*-plane could be due to the microstrip feed line printed directly below the ground plane (along the *y-*axis) has some effect and also caused by the feed connector. Even with slight disruption, the proposed antenna is characterized by a stable radiation pattern within the operating band. In fact, for all the parameters, the discrepancy between simulation and measurement is mainly due to the fabrication tolerance and is also caused by the effect of the feeding cable as the antenna is compact.

## 6. Conclusions

A flexible material substrate has been prepared and characterized in this paper. The realized antenna fabricated on a novel proposed material, for the validation as a substrate. Experimental results exhibit that the realized antenna could achieve an impedance bandwidth from 5.4 to 9.2 GHz with a maximum gain of 4.99 dBi. According to the best of our knowledge, an average radiation efficiency, which is above 80% has been achieved over the operating frequency range, is the highest reported to date for a flexible substrate material of this type. Low dielectric constant and low loss tangent of the material help to improve the antenna performances, whereas optimized design facilitates simple and miniaturized antenna with large bandwidth. The design of the proposed material substrate is simple, easy to fabricate, and compatible with the microwave circuitry. Eventually our objective to design a flexible material substrate for microwave device application with large bandwidth, highly efficient, and adequate peak gain for microwave wireless communication, operating above 6 GHz.
